# Shikonin Directly Targets Mitochondria and Causes Mitochondrial Dysfunction in Cancer Cells

**DOI:** 10.1155/2012/726025

**Published:** 2012-10-15

**Authors:** Benjamin Wiench, Tolga Eichhorn, Malte Paulsen, Thomas Efferth

**Affiliations:** ^1^Department of Pharmaceutical Biology, Institute of Pharmacy and Biochemistry, Johannes Gutenberg University, Staudinger Weg 5, 55128 Mainz, Germany; ^2^Cytometry Core Facility, Institute of Molecular Biology, Ackermannweg 4, 55128 Mainz, Germany

## Abstract

Chemotherapy is a mainstay of cancer treatment. Due to increased drug resistance and the severe side effects of currently used therapeutics, new candidate compounds are required for improvement of therapy success. Shikonin, a natural naphthoquinone, was used in traditional Chinese medicine for the treatment of different inflammatory diseases and recent studies revealed the anticancer activities of shikonin. We found that shikonin has strong cytotoxic effects on 15 cancer cell lines, including multidrug-resistant cell lines. Transcriptome-wide mRNA expression studies showed that shikonin induced genetic pathways regulating cell cycle, mitochondrial function, levels of reactive oxygen species, and cytoskeletal formation. Taking advantage of the inherent fluorescence of shikonin, we analyzed its uptake and distribution in live cells with high spatial and temporal resolution using flow cytometry and confocal microscopy. Shikonin was specifically accumulated in the mitochondria, and this accumulation was associated with a shikonin-dependent deregulation of cellular Ca^2+^ and ROS levels. This deregulation led to a breakdown of the mitochondrial membrane potential, dysfunction of microtubules, cell-cycle arrest, and ultimately induction of apoptosis. Seeing as both the metabolism and the structure of mitochondria show marked differences between cancer cells and normal cells, shikonin is a promising candidate for the next generation of chemotherapy.

## 1. Introduction

Cancer is a leading cause of death worldwide and the global burden of cancer continues to increase, largely because of the growth and aging of the world population [1]. Resistance to cell death and reprogramming of metabolic pathways are two hallmarks of human cancer cells as well as major causes of chemotherapy inefficacy [2]. Mitochondria are key structures for both these traits: (i) mitochondria are crucial for cellular energy production and cell survival; (ii) mitochondria are major regulators in the intrinsic apoptotic pathway [3]. Mitochondrial membrane permeabilization (MMP) and the subsequent release of mitochondrial death effectors (e.g., cytochrome c) are key events for caspase activation and apoptosis [4]. The induction of mitochondrial apoptosis can be triggered by various intracellular stimuli such as Ca^2+^ overload or high levels of reactive oxygen species (ROS) [5]. Furthermore, both these stimuli reinforce each other, leading to Ca^2+^/ROS-mediated mitochondrial dysfunction [6]. 

In cancer cells, the structure and function of mitochondria differ significantly from normal eukaryotic cells [7]. Cancer cells display decreased mitochondrial activity and instead shift to aerobic glycolysis for ATP production, a phenomenon known as the Warburg effect [8]. Cancer cells are often more resistant to activation of the mitochondrial apoptotic pathway due to overexpression of antiapoptotic Bcl-2 family proteins [9] or stabilization of the mitochondrial membrane against apoptosis-associated permeabilization [10]. Another trait associated with cancer cells is elevated ROS levels, probably caused by mitochondrial dysfunction [11]. Therefore, it is conceivable that cancer cells have a lower tolerance to further oxidative insults induced by ROS-generating drugs [9, 12]. Because of the altered mitochondrial functions in neoplasia, direct targeting of mitochondria in cancer cells has become an attractive strategy in cancer chemotherapy over the last few years. A direct induction of apoptosis in cancer cells via the mitochondrial pathway allows one to circumvent upstream signal transduction steps frequently impaired in human cancers [12]. Thus, compounds targeting mitochondria may help to improve the poor results of traditional therapies and furthermore represent a promising approach for the treatment of cancer cells resistant to standard chemotherapy [5].

The naphthoquinone pigment shikonin is the most important pharmacologically active substance in the dried root of *Lithospermum erythrorhizon*. In traditional Chinese medicine root extracts of *Lithospermum erythrorhizon* have been used to treat macular eruption, measles, sore throat, carbuncles, and burns [13]. The antitumor effect of shikonin was first evidenced by its activity against murine sarcoma-180 [14]. A clinical trial using shikonin in 19 cases of late-stage lung cancer revealed that a shikonin-containing mixture was safe and effective for the treatment of late-stage cancer [15]. The mechanism by which shikonin triggers its cytotoxic effect against malignant cells is controversial. A very recent study showed that shikonin inhibits cancer cell glycolysis by targeting tumor pyruvate kinaseM2 [16]. 

In this study, we show for the first time that the natural compound shikonin directly targets mitochondria causing mitochondrial dysfunction and ultimately apoptosis. We confirmed a number of recent findings regarding the cellular effects of shikonin, but our data suggests that most of them are downstream events. The primary effect of shikonin is the direct targeting of mitochondria, which causes a dose-dependent overproduction of ROS and an increase in intracellular calcium levels, leading to breakdown of the mitochondrial membrane potential and induction of the mitochondrial pathway of apoptosis. The increase in intracellular ROS levels and the mitochondrial injury cause other cellular effects such as oxidative DNA damage and inhibition of cancer cell migration.

## 2. Materials and Methods

### 2.1. Chemicals

Shikonin was purchased from Enzo Life Sciences (Lausen, Switzerland) and a 50 mM stock solution was prepared by dissolving it in DMSO. Doxorubicin and daunorubicin were provided by the University Medical Center of the Johannes Gutenberg University (Mainz, Germany) and dissolved in PBS (Invitrogen, Germany) at a concentration of 10 mM. Geneticin was purchased from Sigma-Aldrich (Munich, Germany) at a concentration of 50 mg/mL in sterile-filtered H_2_O.

### 2.2. Cell Cultures

U937, 789-O, MCF-7, SK-BR-3, SW1116, HCT-116, SW680, Capan1, and SUIT-2 cell lines were obtained from the German Cancer Research Center (DKFZ, Heidelberg, Germany). The original source of these cell lines is the American Type Culture Collection (ATCC, USA). U937 and 786-O cells were maintained in complete RPMI 1640 medium with 2 mM L-glutamine (Invitrogen, Germany) supplemented with 10% FBS (Invitrogen, Germany) and 1% penicillin (100 U/mL)-streptomycin (100 *μ*g/mL) (Invitrogen, Germany). MCF-7, SKBR3, SW-1116, HCT-116, SW680, Capan1, and SUIT-2 cells were cultured in complete DMEM culture medium with GlutaMAX (Invitrogen, Germany) supplemented with 10% FBS and 1% penicillin (100 U/mL)-streptomycin (100 *μ*g/mL). 

CCRF-CEM and CEM/ADR5000 cells were kindly provided by Dr. Axel Sauerbrey (University of Jena, Department of Pediatrics, Jena, Germany) and HL-60 and HL60/AR cells by Dr. J. Beck (University of Greifswald, Department of Pediatrics, Greifswald, Germany). The original source of these cell lines is the ATCC. Cells were cultured in complete RPMI 1640 medium with 10% FBS and 1% penicillin-streptomycin. To maintain the multidrug-resistance phenotypes, the MDR1-expressing CEM/ADR5000 cells were treated with 5000 ng/mL doxorubicin and the MRP1-expressing HL-60/AR subline was cultured in medium containing 100 nM daunorubicin. 

MDAMB231pcDNA3 and MDAMB231BCRP breast cancer cell lines transduced with control vector (MDA-MB-231-pcDNA3) or with cDNA for the breast cancer-resistant protein BCRP (MDA-MB-231-BCRP clone 23) were kindly provided by Dr. Douglas D. Ross (University of Maryland, Department of Medicine, Baltimore, Maryland). Both cell lines were continuously treated with 800 ng/mL geneticin. The multidrug resistance profile of these cell lines has been reported [17].

The human osteosarcoma cell line U2OS is stably transfected with a GFP fusion construct of *α*-tubulin. The cell line was kindly provided by Joachim Hehl, Light Microscopy Centre, ETH Zurich. Retinal pigment epithelial (RPE-1) cells stably expressing GFP-EB3 were obtained from Professor W. Krek, ETH Zurich [18]. U2OS-GFP-*α*Tubulin and RPE-1-GFP-EB3 cells were maintained in DMEM medium containing 10% FBS (Invitrogen, Germany) and 1% penicillin (100 U/mL)-streptomycin (100 *μ*g/mL) (Invitrogen, Germany) and were continuously treated with 250 *μ*g/mL and 500 *μ*g/mL geneticin, respectively.

All cell lines were maintained in a humidified environment at 37°C with 5% CO_2_ and subcultured twice per week. All experiments were performed on cells in the logarithmic growth phase.

### 2.3. Resazurin Reduction Assay

Resazurin reduction assay [19] was performed to assess cytotoxicity of shikonin toward various sensitive and resistant cancer cell lines. The assay is based on reduction of the indicator dye, resazurin, to the highly fluorescent resorufin by viable cells. Nonviable cells rapidly lose the metabolic capacity to reduce resazurin and thus produce no fluorescent signal. Briefly, adherent cells were detached by treatment with 0.25% trypsin/EDTA (Invitrogen, Germany) and an aliquot of 1 × 10^4^ cells was placed in each well of a 96-well cell culture plate (Thermo Scientific, Germany) in a total volume of 200 *μ*L. Cells were allowed to attach overnight and then were treated with different concentrations of shikonin. For suspension cells, aliquots of 2 × 10^4^ cells per well were seeded in 96-well-plates in a total volume of 100 *μ*L. Shikonin was immediately added in varying concentrations in an additional 100 *μ*L of culture medium to obtain a total volume of 200 *μ*L/well. After 24 h or 48 h, 20 *μ*L resazurin (Sigma-Aldrich, Germany) 0.01% w/v in ddH_2_O was added to each well and the plates were incubated at 37°C for 4 h. Fluorescence was measured on an Infinite M2000 Proplate reader (Tecan, Germany) using an excitation wavelength of 544 nm and an emission wavelength of 590 nm. Each assay was done at least two times, with six replicates each. The viability was evaluated based on a comparison with untreated cells. IC_50_ values represent the shikonin concentrations required to inhibit 50% of cell proliferation and were calculated from a calibration curve by linear regression using Microsoft Excel.

### 2.4. Microarray Gene Expression Profiling

Total RNA from U937 cells after 24 h of treatment with shikonin at IC_50_ concentration was isolated using RNeasy Kit from Qiagen (Hilden, Germany) according to the manufacture's instruction. The quality of total RNA was checked by gel analysis using the total RNA Nanochip assay on an Agilent 2100 Bioanalyzer (Agilent Technologies GmbH, Berlin, Germany). Only samples with RNA index values greater than 9.3 were selected for expression profiling. Biotin-labeled cRNA samples for hybridization on Illumina Human Sentrix-HT12 Bead Chip arrays (Illumina, Inc.) were prepared according to Illumina's recommended sample labeling procedure based on the modified Eberwine protocol [20]. Biotin-16-UTP was purchased from Roche Applied Science (Penzberg, Germany). The cRNA was column purified with TotalPrep RNA Amplification Kit and eluted in 60 *μ*L of water. Quality of cRNA was controlled using the RNA Nanochip assay on an Agilent 2100 Bioanalyzer and spectrophotometrically quantified (NanoDrop). Hybridization was also performed according the manufacturer's recommendations. Microarray scanning was done using a Beadstation array scanner, setting adjusted to a scaling factor of 1 and PMT settings at 430. Data was extracted for each bead individually, and outliers were removed when the MAD (median absolute deviation) was greater than 2.5. Data analysis was performed by normalizing the signals using the quantile normalization algorithm without background subtraction, and differentially regulated genes were defined by calculating the standard deviation differences of a given probe in a one-by-one comparison of samples or groups. The expression data obtained was filtered with Chipster data analysis platform. Filtered genes were fed into Ingenuity Pathway Analysis software, which assigned them to networks, functions, and pathways.

### 2.5. Real-Time Reverse Transcription-PCR

The same RNA samples used in the microarray experiments were also used for RT-PCR experiments. Total RNA samples were converted to cDNA by reverse transcriptase (Invitrogen) with random hexamer primers. Quantification of cDNA was performed by real-time PCR using a Taq-polymerase master mix (Roche) containing the fluorescent dye SYBR Green (Biozol) and the CFX384 Real-Time PCR Detection System (Bio-Rad). The efficiency of all primer pairs used for real-time PCR expression was better than 90%. PCR was performed with an initial denaturation at 95°C for 5 min followed by 50 cycles consisting of strand separation at 95°C for 30 s and annealing and extension at 60°C for 40 s. After PCR product amplification, melting curves were computed. Expression levels were normalized to the transcription level of G6PD. All samples were run in triplicates.

### 2.6. Fluorescence Scan

Shikonin was solved in aqueous buffer at a final concentration of 50 *μ*M. A 2D excitation (200–600 nm) versus emission (300–700 nm) scan was performed on a Fluorolog-2 spectrofluorometer (Horiba, Unterhaching).

### 2.7. Measurement of Cellular Uptake by Flow Cytometry

Cellular drug uptake experiments were performed according to a recently published protocol [21] using the intrinsic fluorescence of shikonin. Briefly, 1 × 10^6^ U937 cells in complete RPMI 1640 culture medium lacking phenol red indicator were transferred into a 5 mL FACS tube (Becton-Dickinson, Germany) in a total volume of 2 mL. The cells were measured on an LSR-Fortessa FACS analyzer (Becton-Dickinson, Germany) with 640 nm excitation (40 mW) and detected using a 730/45 nm bandpass filter. The background fluorescence of the cells was measured for 2 min and adjusted to be 10×above the electronic noise of the analyzer to ensure precise measurements even in samples with large cellular-based coefficients of variation (CV). Dead cells were excluded by FSC/SSC gating. After 2 min of recording, the FACS tube was removed from the flow cytometer without stopping the recording and shikonin was added immediately in the desired concentration. The cell suspension containing the drug was gently but thoroughly mixed and reinserted into the flow cytometer within 30 sec after removing it from the machine. The measurements continued to 20 min total time at a flow rate of 50–70 cells per second while recording the area signal of all significant channels. Cytographs were analyzed using FlowJo software (Celeza, Switzerland).

### 2.8. Cellular Localization via Confocal Microscopy

For intracellular localization studies of shikonin, cells were stained with MitoTracker GreenFM (Invitrogen, Germany). This fluorescent dye passively diffuses across the plasma membrane and accumulates in active mitochondria. Briefly, 4 × 10^4^ U937 cells were placed in each well of a sterile ibiTreat *μ*-slide (ibidi, Germany) pretreated with polyethylenimine for sufficient cell adhesion or 2 × 10^4^ SKBR3 cells were seeded in untreated ibiTreat *μ*-slides and allowed to attach overnight. According to the manufacturer's protocol, cells were incubated with 100 nM MitoTracker Green FM for 45 min at 37°C in the dark. After incubation, the staining solution was removed and the cells were resuspended in fresh medium containing 25 *μ*M shikonin. Live cell imaging was performed on a Leica TCS SP5 confocal microscope with a 40×/1.30 oil objective (Leica, Germany). The microscope was controlled by LAS AF software (Leica, Germany). A 561 nm laser was used for excitation of shikonin, and the emitted signal was detected at 680–780 nm. MitoTracker Green FM was excited with a 488 nm laser and detected at 500–549 nm. Analysis and averaging of images were performed with LAS AF software; further image processing was carried out with Adobe Photoshop. 

### 2.9. Analysis of Mitochondrial Membrane Potential

The effects of shikonin on the mitochondrial membrane potential were analyzed by JC-1 (Biomol, Germany) staining. JC-1 is a dye that can selectively enter into mitochondria and exhibits an intense red fluorescence in healthy mitochondria with normal membrane potentials. In cells with reduced mitochondrial membrane potential, the red fluorescence disappears. Briefly, 1 × 10^6^ U937 cells were incubated with JC-1 staining solution according to the manufacturer's protocol for 30min. Stained cells were treated with different concentrations of shikonin or DMSO (solvent control) for 6 h. Subsequently, cells were measured in an LSR-Fortessa FACS analyzer (Becton-Dickinson, Germany). For each sample, 1 × 10^4^ cells were counted. The JC-1 signal was measured with 561 nm excitation (150 mW) and detected using a 586/15 nm bandpass filter. The shikonin signal was analyzed with 640 nm excitation (40 mW) and detected using a 730/45 nm bandpass filter. All parameters were plotted on a logarithmic scale. Cytographs were analyzed using FlowJo software (Celeza, Switzerland). All experiments were performed at least in triplicate.

### 2.10. Measurement of Reactive Oxygen Species by Flow Cytometry

2′,7′-Dichlorodihydrofluorescein diacetate (H_2_DCFH-DA) (Sigma-Aldrich, Germany) is a probe used for the highly sensitive and quantifiable detection of reactive oxygen species (ROS). The nonfluorescent H_2_DCFH-DA diffuses into the cells and is cleaved by cytoplasmic esterases into 2′,7′-dichlorodihydrofluorescein (H_2_DCF) which is unable to diffuse back out of the cells. In the presence of hydrogen peroxide, H_2_DCF is oxidized to the fluorescent molecule dichlorofluorescein (DCF) by peroxidases. The fluorescent signal emanating from DCF can be measured and quantified by flow cytometry, thus providing an indication of intracellular ROS concentration [22, 23]. Briefly, 2 × 10^6^ U937 cells were resuspended in PBS and incubated with 2 *μ*M H_2_DCFH-DA for 20 min in the dark. Subsequently, cells were washed with PBS and resuspended in RPMI 1640 culture medium containing different concentrations of shikonin or DMSO (solvent control). After 1 h of incubation, cells were washed and suspended in PBS. Subsequently cells were measured in a FACS Calibur flow cytometer (Becton-Dickinson, Germany). For each sample 1 × 10^4^ cells were counted. DCF was measured at 488 nm excitation (25 mW) and detected using a 530/30 nm bandpass filter. All parameters were plotted on a logarithmic scale. Cytographs were analyzed using FlowJo software (Celeza, Switzerland). All experiments were performed at least in triplicate.

For the measurement of real-time kinetics of ROS induction, U937 cells were stained with H_2_DCFH-DA as described above. The cells were measured on an LSR-Fortessa FACS analyzer (Becton-Dickinson, Germany) with 488 nm excitation (100 mW) for DCF and 649 nm excitation for shikonin. DCF and shikonin fluorescence were detected using a 530/30 nm and a 730/45 nm bandpass filter, respectively. After 2 min of recording, 0.6 *μ*M shikonin was added to the cell suspension and the tube was immediately reinserted into the flow cytometer. The measurement continued up to 1 h total time at a flow rate of 50–70 cells per second. Cytographs were analyzed using FlowJo software (Celeza, Switzerland).

### 2.11. DNA Damage Detection and Quantification by Alkaline Elution Assay

The alkaline elution assay was originally described by Kohn et al. [24] and modified by Epe et al. [25]. In this study, the assay was used to quantify different types of DNA modifications generated in U937 cells after shikonin treatment. Briefly, 10^6^ cells were treated with 0.3 *μ*M shikonin (IC_50_) for 3h and 6 h or DMSO (solvent control). After incubation, cells were lysed on a polycarbonate filter (2 mm pore size) by pumping a lysis solution (100 mM glycine, 20 mM Na_2_EDTA, 2% SDS, 500 mg/L proteinase K, pH 10.0) through the filter for 90 min at 25°C. After washing, the DNA remaining on the filter was incubated with the repair endonuclease Fpg protein (1 *μ*g/mL) for 30 min at 37°C to detect DNA modifications sensitive to oxidative stress. To quantify DNA single-strand breaks, the incubation was also carried out without endonuclease. After Fpg endonuclease incubation the DNA was eluted with an alkaline solution at 25°C and its elution rate determined. The slopes of elution curves obtained with *γ*-irradiated cells were used for calibration (6 G*γ* = 1 single-strand break/10^6^ bp). 

### 2.12. Measurement of Intracellular Calcium Signaling

For analysis of intracellular calcium signaling after shikonin treatment, the chemical calcium indicator indo-1 (Invitrogen, Germany) was used. The dye can be excited in the UV range at ~350 nm and peak emission occurs at ~405 nm and ~485 nm in the Ca^2+^ bound and free states, respectively [26]. In this way, a relatively accurate measurement of the intracellular Ca^2+^ concentration by a fluorometric ratio technique is possible. Briefly, 2 × 10^6^ U937 cells in 2 mL 1640 RPMI culture medium without phenol red indicator were incubated with 1 *μ*M indo-1 for 30 min at 37°C in the dark. Following incubation, cells were centrifuged and resuspended in fresh culture medium. Subsequently, cells were measured on an LSR-Fortessa FACS analyzer (Becton-Dickinson, Germany) with 355 nm excitation (20 mW) and detected using 405/20 nm and 530/30 nm bandpass filters. The ratio of both signals (405/20 nm/530/30 nm) was used as an index for intracellular calcium concentration. After 2 min, the FACS tube was removed from the flow cytometer without stopping the recording and shikonin was added immediately in the desired concentration. The cell suspension containing the drug was mixed and reinserted into the flow cytometer within 30 sec after being removed. The measurements continued up to 60 min total time at a flow rate of 100–150 cells per second while recording the area signal of all significant channels. Cytographs were analyzed using FlowJo software (Celeza, Switzerland). The Ca^2+^ exchanger ionomycin (Sigma-Aldrich, Germany) raises intracellular calcium levels and served as positive control at a final concentration of 0.4 *μ*M. Cells treated with DMSO served as a solvent control.

### 2.13. Cell Cycle Analysis

For cell-cycle analysis, 1 × 10^6^ U937 cells were treated with different concentrations of shikonin for 24 h. Following incubation, cells were washed in PBS and fixed in ice-cold 95% ethanol (Sigma-Aldrich, Germany). After washing in PBS, cells were incubated with 10 *μ*g/mL RNaseA (Applichem Lifescience, Germany) and 50 *μ*g/mL propidium iodide (PI, Sigma-Aldrich, Germany) in PBS for 1 h in the dark. Cells were measured on an LSR-Fortessa FACS analyzer (Becton-Dickinson, Germany). 1 × 10^4^ cells were counted for each sample. PI was measured with 488 nm excitation (100 mW) and detected using a 610/20 nm bandpass filter. Cytographs were analyzed using FlowJo software (Celeza, Switzerland). All experiments were performed at least in triplicate.

### 2.14. Caspase-Glo 3/7 and Caspase-Glo 9 Assay

The influence of shikonin on caspase 3/7 and caspase 9 activity in U937 leukemia cells was detected using Caspase-Glo 3/7 and Caspase-Glo 9 Assay kits (Promega, Germany). Cells cultured in RPMI 1640 medium were seeded in 96-well plates and treated with different concentrations of shikonin or DMSO (solvent control). After 6 h of treatment, cellular caspase3/7 or caspase9 activity was determined according to the manufacturer's protocol. Luminescence was measured using the Infinite M2000 Proplate reader (Tecan, Germany). Caspase activity was expressed as percentage of the untreated control.

### 2.15. Scratch Migration Assay

The scratch migration assay is a well-developed method to investigate drug effects on cell migration *in vitro *[27]. Briefly, 1 × 10^6^ SKBR3 cells were seeded in each well of a 6-well plate and allowed to grow to a confluent monolayer. The cell monolayer was carefully scraped with a sterile p200 pipet tip to create a scratch. Subsequently, cells were washed with PBS and treated with DMEM culture medium containing different subtoxic concentrations of shikonin or DMSO (solvent control). Images of the scratches were taken every 3 h using a phase contrast microscope (Optika, Italy) at 10x magnification. Data analysis was performed with TScratch software [28].

### 2.16. Imaging of the Structure and Dynamics of the Microtubule Cytoskeleton by Confocal Microscopy

Live cell imaging was performed on a Leica TCS SP5 confocal microscope with a 40x/1.30 oil objective. The microscope was controlled by LAS AF software. A 488 nm laser was used for excitation of GFP, and the emitted signal was detected at 500–549 nm. Shikonin was excited with a 561 nm laser and the emitted fluorescence was detected at 680–780 nm. Analysis and averaging of images were performed with LAS AF software; further image processing was carried out with Adobe Photoshop.

2 × 10^4^ U2OS-GFP-*α*Tubulin or RPE-1-GFP-EB3 cells were seeded in each well of a sterile ibi Treat *μ*-slide (ibidi, Germany) and cells were allowed to attach overnight. Cells were treated with 25 *μ*M shikonin and subsequently analyzed by confocal microscopy. Each experiment was repeated at least three times and representative images and videos were selected.

## 3. Results

### 3.1. Cytotoxic Effect of Shikonin on Cancer Cells

To investigate the effect of shikonin against various types of cancer, a panel of 15 sensitive and multidrug resistant cancer cell lines was treated with shikonin for 24 or 48 h. The results are summarized in Table 1. Shikonin inhibited proliferation by 50% in nearly all cancer cell lines at concentrations below 10 *μ*M after 24 h. Only the pancreatic carcinoma cell line SUIT2 was more resistant to shikonin; it showed IC_50_ values of 12.9 and 18.5 *μ*M after 24 and 48 h of treatment, respectively. Interestingly, all five tested leukemia cell lines had IC_50_ values below 1 *μ*M, and the most sensitive cell line was the histiocytic leukemia cell line U937, which was subsequently used for gene expression analysis under shikonin treatment.

Besides non-MDR cell lines like U937, shikonin appeared to be highly effective against three known multidrug-resistant cancer cell lines: CEM/ADR5000, HL-60/AR, and MDAMB231/BCRP (Table 1). Similarly to their sensitive counterparts, these cell lines showed no or negligible resistance against shikonin.

### 3.2. Gene Expression Profiling Identifies Novel Molecular Key Players and Genetic Networks

We performed gene expression analysis to identify possible targets and mechanisms of shikonin's anticancer activities in histocytic leukemia U937 cells. U937 cells were treated with 0.3 *μ*M (IC_50_) shikonin or DMSO solvent control for 24 h before total RNA was isolated for a whole human genome mRNA gene expression microarray. Bioinformatic analysis identified 683 genes significantly deregulated (*P* < 0.01) after shikonin treatment. For a number of genes, the expression array was validated using RT-qPCR, which yielded comparable results (See Table S1 in Supplementary Material available online at doi: 10.1155/2012/726025). Using the Ingenuity Pathway Analysis tool, we correlated the deregulated genes with 71 biological functions (or diseases) including cell death and cell cycle, cancer, cellular movement and cell morphology, inflammatory response, protein folding, synthesis and posttranscriptional modification, energy production and cellular growth, and DNA repair and free radical scavenging. Furthermore, four genetic networks were found to be significantly deregulated in U937 cells after shikonin treatment and were correlated to discrete cellular functions like DNA repair, energy production, cell morphology, and cellular development. Subsequent analysis of the microarray showed that a whole subset of genes responsible for mitochondrial function was deregulated. Combining the information obtained from Ingenuity Pathway Analysis and our own in-depth analysis of the deregulated genes, we were able to divide the major cellular functions affected by shikonin into four categories: mitochondrial function, ROS induction and DNA damage, apoptosis and cell cycle arrest, and cytoskeleton and migration (Figure S1).

### 3.3. Mitochondrial Drug Accumulation and Breakdown of the Mitochondrial Membrane Potential

**Figure 1:**
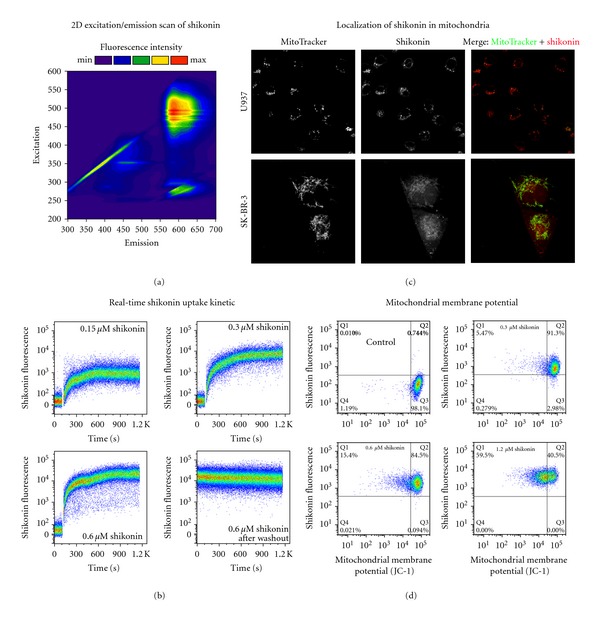
Shikonin accumulates in mitochondria and causes a breakdown of the mitochondrial membrane potential. (a) 2D excitation (200–600 nm) versus emission (300–700 nm) fluorescence spectrum of 50 *μ*M shikonin in aqueous buffer. (b) Real-time kinetics and quantification of cellular shikonin uptake by flow cytometry. The inherent fluorescence of intracellular shikonin was measured at 640 nm excitation with a 730/45 nm bandpass filter. A dose-dependent increase of the cellular shikonin fluorescence was observed after treatment with increasing concentrations (0.15, 0.3 and 0.6 *μ*M) of shikonin. After 20 min of incubation with 0.6 *μ*M shikonin and subsequent washing of the cells, shikonin's fluorescence was still detectable in cells, indicating persistent intracellular accumulation of shikonin. (c) Shikonin localizes to mitochondria. U937 or SK-BR-3 cells were stained with MitoTracker Green and subsequently treated with 25 *μ*M shikonin. Cells were then examined by confocal microscopy at an excitation wavelength of 488nm and 561 nm and emission at 500–549 nm and 680–780 nm for MitoTracker Green and shikonin, respectively. (d) Breakdown of the mitochondrial membrane potential. U937 cells were stained with JC-1, which has a strong red fluorescence in healthy mitochondria. Shikonin induced a dose-dependent decrease of the red JC-1 fluorescence after 6 h of treatment with increasing concentrations of shikonin (0.3, 0.6, and 1.2 *μ*M), indicating a breakdown of the mitochondrial membrane potential.

Since shikonin had a strong effect on gene expression associated with mitochondrial function and metabolism, we proposed that the mitochondrion itself is a possible target of the compound. Shikonin has a specific inherent fluorescence spectrum that has until now remained either unnoticed or ignored. We measured a two-dimensional fluorescence spectrum of emission versus excitation wavelengths for shikonin and observed strong and specific fluorescence in the visible spectrum (Figure 1(a)). We performed flow cytometric cellular uptake assays based on the inherent fluorescence of the molecule, which indicate that shikonin enters the cells within 10 min and that the cellular concentration increases with higher application doses (Figure 1(b)). Washout experiments showed that shikonin remains stable inside the cells for at least 20 min, suggesting that no direct diffusion or transport out of the cell takes place (Figure 1(b), lower right panel). We further exploited the inherent fluorescence of shikonin to map its cellular localization in U937 and SK-BR-3 cells by confocal fluorescence microscopy. This examination revealed tubular structures with strong shikonin fluorescence, likely to be mitochondria, around the nucleus. In order to validate this assumption, U937 cells were stained with the mitochondrial dye MitoTracker Green and then incubated with a higher dose of shikonin to enhance detection via confocal microscopy. As shown in Figure 1, shikonin (Figure 1(c), middle position) was clearly colocalized with the MitoTracker signal (Figure 1(c), left position) only minutes after shikonin application, corroborating the fact that shikonin accumulates directly in and around the mitochondria after its rapid cellular uptake. For improved visualization of the mitochondria, the experiment was repeated with the adherent SKBR-3 breast cancer cell line, resulting in a similar accumulation of shikonin in and around the mitochondria (Figure 1(c)). 

The localization of shikonin to the mitochondria naturally led to the question of whether shikonin indeed influences mitochondrial function as suggested by our gene expression profiling results. We hypothesized that shikonin negatively influences the mitochondrial membrane potential. U937 cells were stained with JC-1, a marker for intact mitochondrial membrane potential, and treated with 0.3–1.2 *μ*M shikonin for 6h. Subsequently, the cellular shikonin signal and the JC-1 signal were analyzed by flow cytometry. The potential difference across the mitochondrial membrane was significantly reduced in cells treated with shikonin, as indicated by reduced red fluorescence of JC-1 in these samples (Figure 1(d)). The mitochondrial membrane potential was reduced more quickly and to a greater extent in cells treated with increasing doses of shikonin (data not shown). This suggests that shikonin itself acts to reduce the membrane potential rather than interacting with or blocking a specific target protein.

### 3.4. Induction of Reactive Oxygen Species and DNA Damage

**Figure 2:**
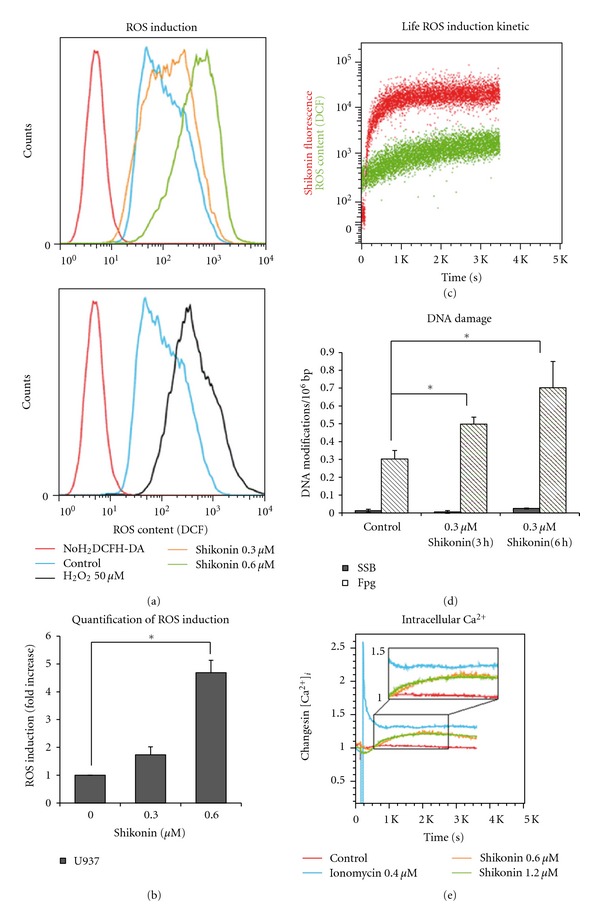
Induction of ROS, oxidative DNA damage, and elevated intracellular Ca^2+^ levels by shikonin. (a) Flow cytometric analysis of ROS levels after treatment with different concentrations of shikonin for 1 h or 50 *μ*M H_2_O_2_ for 15 min in living U937 cells. Cells were stained with H_2_DCFH-DA and measured at 488 nm excitation and detected using a 530/30 nm bandpass filter. (b) Statistical quantification of ROS induction after shikonin treatment in U937 cells. Data points represent mean (fold change) ± SEM of at least three independent experiments. (c) ROS induction kinetics in live cells. U937 cells were stained with H_2_DCFH-DA and ROS induction was measured by flow cytometry. After 2 min, shikonin was added to the cells and measurement was continued for 1 h. Shikonin was excited at 640 nm and detected with a 730/45 nm bandpass filter. DCF was excited with a 488 nm laser and detected using a 530/30 nm bandpass filter. (d) Induction of DNA damage by shikonin measured using alkaline elution technique. Columns indicate the number of DNA single-strand breaks (SSB) and of Fpg-sensitive modifications (oxidative DNA damage) after shikonin treatment. Data points represent mean ± SEM of at least three independent experiments. (e) Real-time kinetics of intracellular Ca^2+^ levels after treatment with different concentrations of shikonin or ionomycin in U937 cells. Cells were stained with indo-1 and [Ca^2+^]_*i*_ was measured by flow cytometry. After 2 min, shikonin was added to the cells and measurement was continued for 1 h. Indo-1 was excited with a 355 nm laser and the ratio of the signals detected using a 405/20 nm filter and a 530/30 filter (405/20 nm/530/30 nm) was used as an index for intracellular calcium concentration (*significant difference according to Student's *t*-test, *P* < 0.05).

As we have shown, shikonin enters cells quickly, accumulates in the mitochondria, and negatively influences the mitochondrial membrane potential. However, we wanted to know whether the cytotoxicity of shikonin is mediated largely by mitochondrial deregulation. Mitochondria are the most significant source of cellular ROS [29]. Given our results, particularly those on shikonin's effect on the mitochondrial membrane potential, we analyzed cellular ROS levels after shikonin treatment by H_2_DCFH-DA staining and flow cytometry. Indeed, we observed a clear dose-dependent increase in cellular ROS levels after very short incubation periods (1 h) with shikonin (Figures 2(a) and 2(b)) confirming previous reports [30]. ROS levels after treatment with 0.6 *μ*M shikonin are comparable to those after incubation with 50 *μ*M H_2_O_2_, our positive control. Thus, shikonin is indeed a potent ROS inducer (Figure2(a)). We performed real-time measurements of shikonin uptake and ROS induction to correlate the kinetics of these two processes. We observed increased ROS production shortly after cellular shikonin uptake, and ROS levels continuously increased for at least 1 h after exposure to shikonin (Figure 2(c)). The kinetics of ROS induction suggest that it is a primary and direct effect of shikonin itself and not a downstream effect mediated indirectly. Subsequent literature research supports our conclusion, revealing that naphthoquinones other than shikonin have been previously implicated in ROS induction via a futile redox cycle in isolated mitochondria [31].

Abnormal accumulation of ROS is likely to give rise to oxidative stress and cause DNA damage by modifications such as 7,8-dihydro-8-oxoguanine (8-oxoG) [32]. Since our results of the gene expression profiling also indicated DNA damage after shikonin treatment, we anticipated that shikonin-induced ROS was the cause of oxidative DNA damage. The alkaline elution technique was used to quantify DNA modifications sensitive to Fpg protein, a repair endonuclease that recognizes and nicks the DNA at sites of oxidative purine modifications such as 8-oxoG. Untreated U937 control cells showed few single-strand breaks, but had a mildly increased level of 0.3oxidative DNA modifications/10^6^ bp (Figure 2(d)). This elevated level of oxidative DNA damage corroborates previous findings showing an increased amount of oxidative stress and DNA damage in leukemia cells [33]. Upon treatment of U937 cells with 0.3 *μ*M shikonin, there was a 1.6-fold and 2.3-fold increase in the amount of oxidative DNA modifications after 3 and 6 h treatment, respectively (Figure 2(d)). Interestingly, there was no significant generation of single-strand DNA breaks observed in cells treated with 0.3 *μ*M shikonin during the short incubation periods (3and 6 h). This observation agrees with a model in which shikonin causes oxidative DNA damage mediated primarily by inducing ROS (Figures 2(a)–2(c)).

### 3.5. Induction of Intracellular Calcium Signaling

Increased intracellular ROS levels are known to disturb cellular calcium signaling [6]. Since we found several genes involved in calcium homeostasis strongly deregulated after shikonin treatment (e.g., genes coding for the calcium binding proteins S100A8 and S100A9), we analyzed the effect of shikonin on the intracellular calcium concentration [Ca^2+^]_*i*_ by indo-1 staining. In contrast to ionomycin, a molecule used to increase [Ca^2+^]_*i*_, treatment with shikonin caused a very short and weak decrease in free [Ca^2+^]_*i*_ that quickly reversed into a slow but continuous increase in [Ca^2+^]_*i*_ in U937 cells (Figure 2(e)). After 30 min of treatment with shikonin, the concentration of intracellular calcium is more than 1.2-fold higher than that in the DMSO treated control cells. Furthermore, the effect is stable for at least 1 h. Interestingly, different concentrations of shikonin (0.6 and 1.2 *μ*M) appear to have the same effect, and no obvious dose dependency could be detected. The slow release of [Ca^2+^]_*i*_ after treatment with shikonin suggests that shikonin does not interact directly with calcium storage regulators or serve as a calcium ionophore/shuttler as does ionomycin. Instead, it is likely that shikonin induces release of [Ca^2+^]_*i*_ by the well-known ROS-calcium signaling pathway [6], which would also explain the rather slow kinetics of calcium signaling observed (Figures 2(c) and 2(e)).

### 3.6. Long-Term Treatment with Shikonin Induces Cell-Cycle Arrest and the Mitochondrial Pathway of Apoptosis

**Figure 3:**
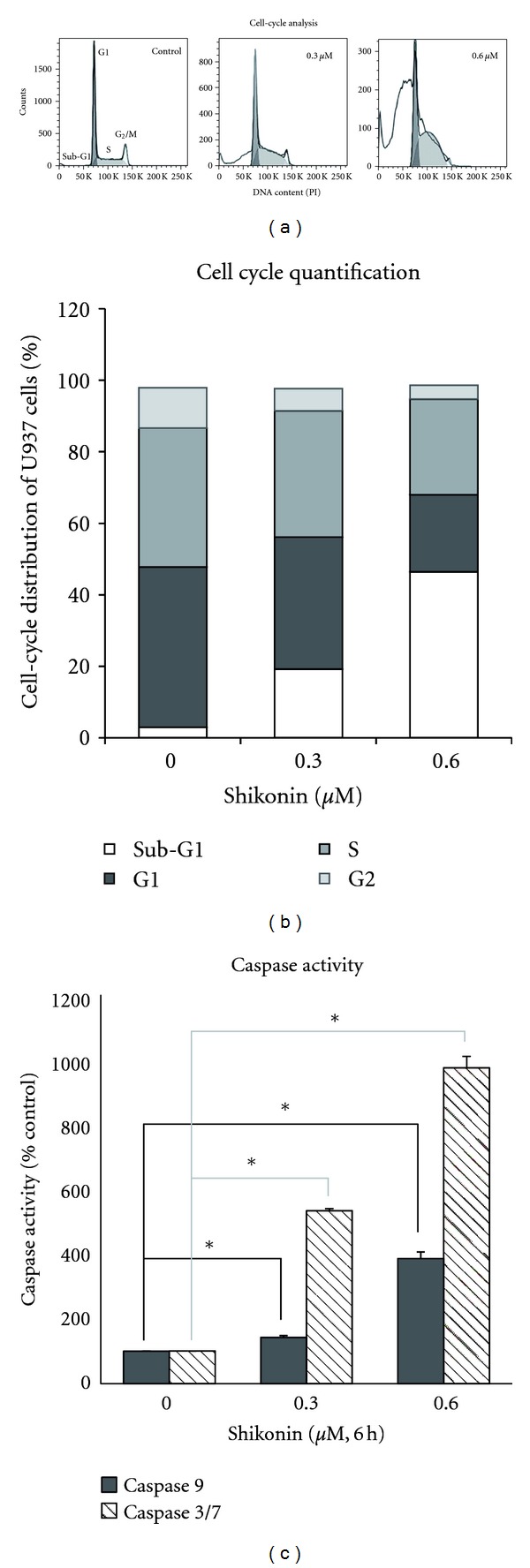
Shikonin induces cell cycle arrest and apoptosis in U937 cells. (a) Typical DNA content histograms of U937 cells treated with increasing concentrations of shikonin for 24 h. (b) Statistical analysis of cell cycle distribution of U937 cells after treatment with different concentrations of shikonin for 24 h. Data points are means of at least three independent experiments. (c) Enzymatic activity of caspase 3/7 and caspase 9 after 6 h of shikonin treatment in U937 cells. The caspase activity (mean ± SD of at least three experiments) is expressed as percentage relative to the untreated control (*significant difference according to Student's *t*-test, *P* < 0.05).

Given the effects of shikonin on ROS generation, oxidative genotoxic stress induction and calcium signaling, we further explored the long-term effects of shikonin on the cell cycle. Flow cytometric cell-cycle analysis was performed on U937 cells after 24 h of treatment with different concentrations of shikonin (Figure 3(a)). Shikonin significantly increased the percentage of cells in the sub-G1 phase in a dose-dependent manner (Figures 3(a) and 3(b)), representing an increase in cell death, possibly by apoptosis [34]. However, the amount of cells in the G1 and S phase was less affected than the G2 cell population at 0.3 *μ*M (IC_50_) shikonin (Figure3(b)). At this concentration, the population of cells in the G2 phase was decreased by nearly half, indicating that, although the cells were still able to enter S phase to some extent, they were significantly stalled in the S phase. Interestingly, the number of cells in the S phase was increased at subtoxic concentrations of 0.1 *μ*M shikonin, suggesting that very low doses of shikonin might activate signaling processes driving cell proliferation (data not shown). In summary, effective doses of shikonin seem to cause an arrest of cells in the G1 and S phase, preventing them from entering G2 phase. Ultimately, this arrest leads to a strong increase in the number of apoptotic cells.

Since shikonin accumulates in the mitochondria and disrupts the mitochondrial membrane potential, we hypothesized that induction of cell death in shikonin-treated cells may be due to activation of the mitochondrial or intrinsic apoptotic pathway leading to caspase 9 activation. Caspase 9 is activated after the release of cytochrome c from the mitochondria, and once activated it cleaves and activates the effector caspases3 and 7, which mediate apoptotic cell death [4, 35]. We analyzed the activation of caspases 3, 7, and 9 in U937 cells after 6 h of treatment with different concentrations of shikonin. We observed a significant dose-dependent increase in the activity of all three caspases in shikonin-treated samples, consistent with the hypothesis that shikonin activates the intrinsic pathway of apoptosis (Figure 3(c)). 

### 3.7. Inhibition of Cancer Cell Migration and Microtubule Dynamics

**Figure 4:**
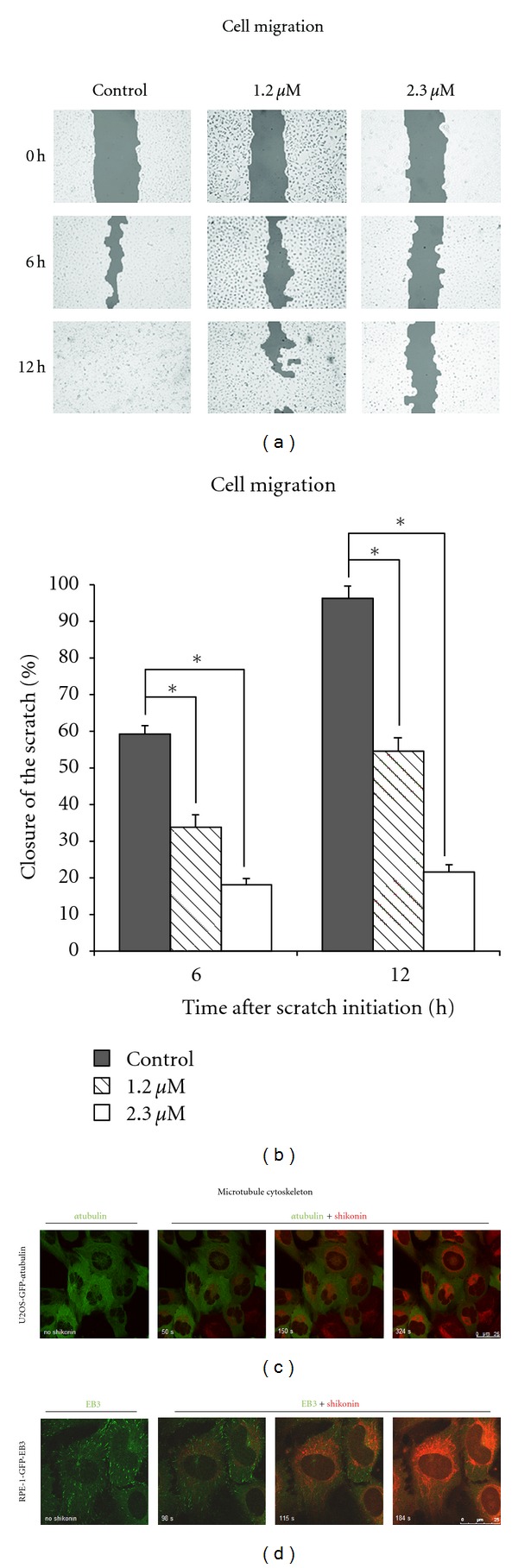
Shikonin inhibits cancer cell migration and affects microtubule structure and dynamics. (a) Typical pictures at 0, 6, and 12 h of scratch migration assays using SK-BR-3 cells treated with different effective, but in this time-frame subtoxic, concentrations of shikonin. (b) Statistical quantification of the scratch migration assay. Data points represent the mean ± SEM of at least three independent experiments. (c) Live imaging of U2OS-GFP-*α*Tubulin cells stably transfected with a GFP fusion construct of *α*-tubulin and treated with 25 *μ*M shikonin. With increasing cellular concentrations of shikonin, the number of distinct tubulin filaments decreased and the tubulin staining became progressively diffuse. (d) Live imaging of RPE-1-GFP-EB3 cells stably expressing GFP-EB3 and treated with 25 *μ*M shikonin. Shikonin caused a slowdown and finally a complete disappearance of EB3 particles within 3min after application, indicating disrupted microtubule formation (*significant difference according to Student's *t*-test, *P* < 0.05).

The initial microarray experiment identified a set of genes sensitive to shikonin and correlated with cytoskeleton formation, cellular movement, and morphology. We speculated that shikonin negatively influences cell motility and performed scratch migration assays using the highly metastatic breast cancer cell line SKBR-3 to investigate the effect of shikonin on cancer cell migration (Figures 4(a) and 4(b)). Experiments with SKBR3 cells treated with DMSO solvent control demonstrated complete scratch closure in most cases after 12 h. However, cells treated with 1.2 *μ*M shikonin (IC_50_/8) showed a considerably delayed closure of the scratch; after 12 h only 55% of the initial scratch width was closed. At a concentration of 2.3 *μ*M shikonin (IC_50_/4), only 22% of the initial scratch width was recolonized after 12 h. These results strongly indicate that shikonin inhibits migration of SK-BR-3 breast cancer cells at sub-toxic concentrations. 

Microtubules are indispensable for the directional migration of cells [36]. Since our gene expression profiling showed a high number of deregulated genes associated with the microtubule cytoskeleton, we presumed that shikonin's effect on microtubules was responsible for its ability to inhibit breast cancer cell migration. We therefore treated U2OS-GFP-*α*Tubulin cells with 25 *μ*M shikonin and analyzed the drug uptake as well as the direct effect of shikonin on the microtubule cytoskeleton in real-time using high-resolution confocal microscopy (Video S1). We observed rapid accumulation of shikonin within the cells' mitochondria, but we detected no colocalization of shikonin with tubulin filaments. However, with increasing cellular concentrations of shikonin, the number of distinct tubulin filaments decreased and the tubulin staining became progressively more diffuse (Figure 4(c)). Due to highly specific accumulation of shikonin in and around the mitochondria, we concluded that the disassembly of the tubulin network is an indirect downstream effect of shikonin. The tubulin filament disassembly could be a consequence of the reduced amount of ATP generated in the mitochondria by oxidative respiration or of the deregulated calcium signaling. 

The microtubule-associated End binding protein-3 (EB3) binds to growing microtubule plus ends. The GFP-EB3 fusion proteins generate a punctuate pattern of EB3-GFP comets throughout the cell and serve as an elegant marker for visualizing microtubule growth events and dynamics [18, 37]. In order to examine variations in microtubule dynamics after shikonin treatment, RPE-1-GFP-EB3 cells were treated with 25 *μ*M shikonin and drug uptake as well as effects on EB3-GFP particle dynamics was analyzed in real-time experiments using high-resolution confocal microscopy (Video S2). As expected, there was no colocalization of shikonin with EB3 comets. However, shikonin treatment did cause a strong slowdown and finally a complete disappearance of EB3 particles within 3 min of application. EB3-GFP particles are known to disappear when microtubule growth is paused or switches from a state of growth into a state of shrinkage [18]. Furthermore, microtubule formation is dependent on sufficient amounts of ATP [38] and is sensitive to changes in calcium levels [39], both of which are significantly affected by shikonin.

## 4. Discussion

We showed that shikonin has a strong cytotoxic effect on a wide variety of cancer cell lines, especially different types of leukemia and several known MDR cell lines. Microarray-based gene expression analysis of U937 leukemia cells suggested that the cytotoxicity of shikonin is based on the disruption of normal mitochondrial function, overproduction of ROS, inhibition of cytoskeleton formation, and finally induction of cell-cycle arrest and apoptosis. We were able to validate all of these effects using *in vitro* cell culture experiments exploiting the specific natural fluorescence of shikonin and thereby identify the possible primary cellular mechanism of shikonin's cytotoxicity. We support our claim with the finding that shikonin immediately accumulates in the mitochondria of cancer cells and disrupts mitochondrial function, as evidenced by the loss of mitochondrial membrane potential. It was recently shown that shikonin induces ROS and apoptosis in cancer cells [30] and our results fully concur with this assertion. However, we would suggest that other previously described mechanisms of action for shikonin such as the induction of necroptosis [40], inhibition of topoisomerase II activity [41], downregulation of NF*κ*B signaling [42], cell-cycle arrest through upregulation of p53 and downregulation of cyclin-dependent protein kinase 4 [43], inhibition of proteasome function [44], inhibition of tumor necrosis factor alpha [45], deregulation of calcium signaling, and microtubule disintegration are actually downstream effects mediated (in most cases) not by a direct interaction of shikonin with the suggested targets but rather by the direct generation of ROS, the subsequent dysregulation of mitochondria and induction of oxidative damage. 

A recent study showed that shikonin interferes with cancer cells' energy generation by targeting tumor pyruvate kinase-M2 and thereby inhibiting glycolysis [16]. Our study confirms that shikonin treatment causes reduced energy production in cancer cells by affecting the mitochondrial membrane potential, but the observed effects of shikonin on ROS and mitochondrial function are not likely to be purely based on blocking glycolysis. If glycolysis is unable to serve as a source of acetyl-CoA for energy generation, cells can compensate by shift to other metabolic pathways such as fatty acid oxidation [46] or glutamine utilization [47]. Our data does not exclude the possibility that shikonin has an effect on pyruvate kinase-M2, but the direct targeting of mitochondria and the complete loss of the mitochondrial membrane potential as well as the rapid induction of ROS make the electron chain the more likely target of shikonin.

Shikonin can be categorized as a mitocan [48], a class of compounds that act by interfering with energy-generating mitochondrial processes, which in turn leads to ROS accumulation, mitochondrial destabilization, and induction of apoptosis [12]. Shikonin itself is a naphthoquinone derivative, and various substituted naphthoquinones have been shown to be capable of redox cycling in isolated mitochondria [31]. During this process, reductive enzymes, for example, mitochondrial NADH-ubiquinone oxidoreductase (complex 1), metabolize quinones to unstable semiquinones through one-electron reduction reactions [49]. When molecular oxygen is present, such semiquinones enter into a redox cycle leading to reformation of the original quinone, with the associated generation of reactive oxygen species. Ultimately, this cycle results in excessive ROS accumulation, depolarization of the mitochondrial membrane, and induction of apoptosis [50]. Due to the quinone structure of shikonin and its accumulation in the mitochondria, we believe that the ROS induction caused by shikonin is also based on such a futile mitochondrial redox cycling. The elevated levels of ROS strain the mitochondria, leading to a breakdown of the mitochondrial membrane potential and finally to the release of proapoptotic compounds and thus the activation of caspases involved in the intrinsic pathway of apoptosis. The oxidative DNA damage detected is also a consequence of the elevated ROS production and could likely be the trigger of the observed cell-cycle arrest [51].

Besides inducing ROS, some quinones have been shown to cause release of calcium from isolated mitochondria [31]. This is consistent with the elevated levels of [Ca^2+^]_*i*_ observed after shikonin treatment. We showed that shikonin, in contrast to ionomycin, caused a slow and continuous increase in intracellular calcium concentrations. This suggests that shikonin does not shuttle extracellular or intracellular stored calcium actively, but rather causes a calcium release from calcium stores or other organelles, for example, mitochondria, by an indirect mode such as via ROS signaling pathway [6]. Nevertheless, elevated levels of [Ca^2+^]_*i*_ and ROS together appreciably disturb normal calcium signaling [6]. Increased calcium levels promote the disassembly of microtubules by direct destabilization of growing microtubule ends [39], which is in accordance with our findings that shikonin inhibits cancer cell migration by the disruption of microtubule cytoskeleton dynamics. Indeed, shikonin treatment results in a complete inhibition of EB3 protein dynamics and a loss of distinct microtubule filaments, suggesting that the ATP shortage and deregulation of calcium levels are dually destructive. These findings motivate further investigations on the effect of shikonin in the treatment of highly invasive cancer types. 

Many established anticancer agents affect upstream signaling pathways that ultimately converge on mitochondria as regulators of cell death and survival [52]. These signaling pathways are often deregulated in human cancers, and for this reason many MDR phenotypes are resistant to classical anticancer agents [2]. Thus, compounds that directly target mitochondria can bypass deregulated upstream signaling events and thereby circumvent the resistance mechanisms of cancer cells [5]. However due to the basic mode of action it is likely that shikonin also has an effect on noncancer cells. Yet, shikonin bypasses resistances of known MDR cell types and this makes further research on better and more direct application methods an interesting project. Numerous animal studies showed that the therapeutic effects of shikonin apparently predominate the side effects [13, 53] and a clinical trial with shikonin showed that it can be utilized in therapy [15]. Future studies should concentrate on the reduction of side effects by chemical derivatization or tissue targeted application.

In summary, our results indicate that shikonin accumulates in the mitochondria of cancer cells, disrupts mitochondrial function, and finally causes apoptosis. As mitochondria generate the majority of the cellular ATP supply and also regulate the cell death machinery, they are promising targets for cancer therapy. Hence, shikonin may have potential for cancer treatment. 

## Supplementary Material

The Supplementary Material contains a comparison of the results of the microarray gene expression and the real-time reverse transcription PCR for six selected genes (Table S1). Shikonin-affected mechanisms in U937 cells detected by gene expression analysis are summarized in Figure S1. Furthermore video recordings showing the effect of shikonin on the microtubule cytoskeleton are provided (Video S1 and Video S2).

## Figures and Tables

**Table 1:** IC_50_ values (mean ± SEM) of shikonin for a panel of 15 different sensitive and resistant cancer cell lines after 24and 48 h as assayed by resazurin reduction assay.

Cell line	Cancer type	Shikonin	
		IC_50_ [*μ*M] (24 h)	IC_50_ [*μ*M] (48 h)
U937	Histiocytic leukemia cell line	0.30 ± 0.003	0.19 ± 0.003
CCRF-CEM	Acute lymphocytic leukemia cell line	0.37 ± 0.01	0.24 ± 0.001
CEM/ADR5000*	Acute lymphocytic leukemia cell line	0.36 ± 0.05	0.38 ± 0.01
HL-60	Acute myelocytic leukemia cell line	0.39 ± 0.01	0.42 ± 0.001
HL-60/AR*	Acute myelocytic leukemia cell line	0.95 ± 0.003	0.47 ± 0.001
MCF-7	Breast carcinoma cell line	9.05 ± 0.08	10.51 ± 0.03
SK-BR-3	Breast adenocarcinoma cell line	9.21 ± 0.08	8.70 ± 0.03
MDA-MB-231/pcDNA3	Breast carcinoma cell line	1.23 ± 0.03	0.88 ± 0.03
MDA-MB-231/BCRP*	Breast carcinoma cell line	2.61 ± 0.06	1.48 ± 0.04
786-O	Kidney carcinoma cell line	9.44 ± 0.13	8.03 ± 0.06
SW-1116	Colorectal carcinoma (GIII) cell line	6.63 ± 0.09	4.42 ± 0.06
HCT-116	Colorectal carcinoma	4.74 ± 0.07	8.54 ± 0.01
SW680	Colorectal carcinoma	7.21 ± 0.13	2.96 ± 0.06
Capan1	Pancreas adenocarcinoma cell line	7.23 ± 0.28	6.21 ± 0.14
SUIT-2	Pancreatic carcinoma cell line	12.92 ± 0.35	18.50 ± 0.09

*MDR cancer cell lines with various drug resistances.

## Abbreviations

8-oxoG: 7,8-Dihydro-8-oxoguanine
[Ca^2+^]_*i*_: Intracellular calcium concentration
CV: Coefficient of variation
DCF: Dichlorofluorescein
FACS: Fluorescence-activated cell sorting
FBS: Fetal bovine serum
Fpg: Formamidopyrimidine DNA glycosylase
GFP: Green fluorescent protein
H_2_DCF: 2′,7′-Dichlorodihydrofluorescein
H_2_DCFH-DA: 2′,7′-Dichlorodihydrofluorescein diacetate
JC-1: 5,5′,6,6′-Tetrachloro-1,1′,3,3′-tetraethyl-benzimidazolylcarbocyanine iodide
MDR: Multiple drug resistance
MMP: Mitochondrial membrane permeabilization
PBS: Phosphate buffered saline
PI: Propidium iodide
ROS: Reactive oxygen species
SEM: Standard error of the mean
SD: Standard deviation
SSB: Single strand breaks.
