# Supplementary material for: Shikonin Directly Targets Mitochondria and Causes Mitochondrial Dysfunction in Cancer Cells
Source: Evid Based Complement Alternat Med. 2012 Oct 15;2012:726025. doi: 10.1155/2012/726025 (PMC3478753; doi:10.1155/2012/726025)
Supplement: Supplementary file 1 — The Supplementary Material contains a comparison of the results of the microarray gene expression and the real-time reverse transcription PCR for six selected genes (Table S1). Shikonin-affected mechanisms in U937 cells detected by gene expression analysis are summarized in Figure S1. Furthermore video recordings showing the effect of shikonin on the microtubule cytoskeleton are provided (Video S1 and Video S2). [file 726025.f1.docx]

Supplementary Table 1:


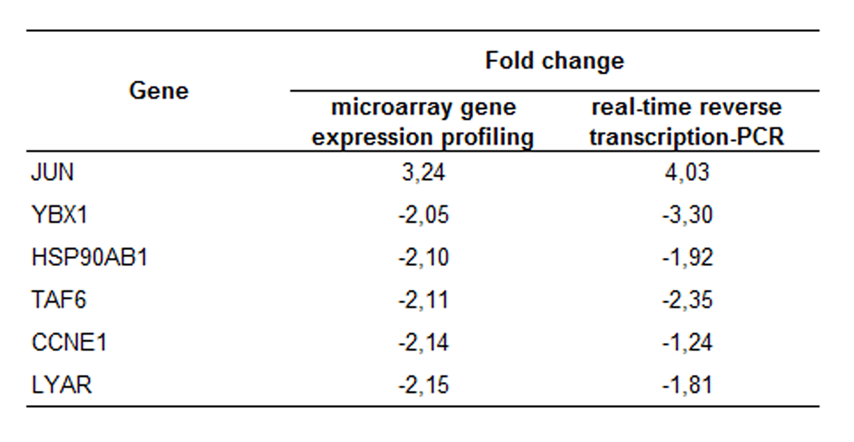


**Supplementary Table 1:** Results of microarray gene expression profiling and real-time reverse transcription-PCR for five selected genes.

Supplementary Figure S1:


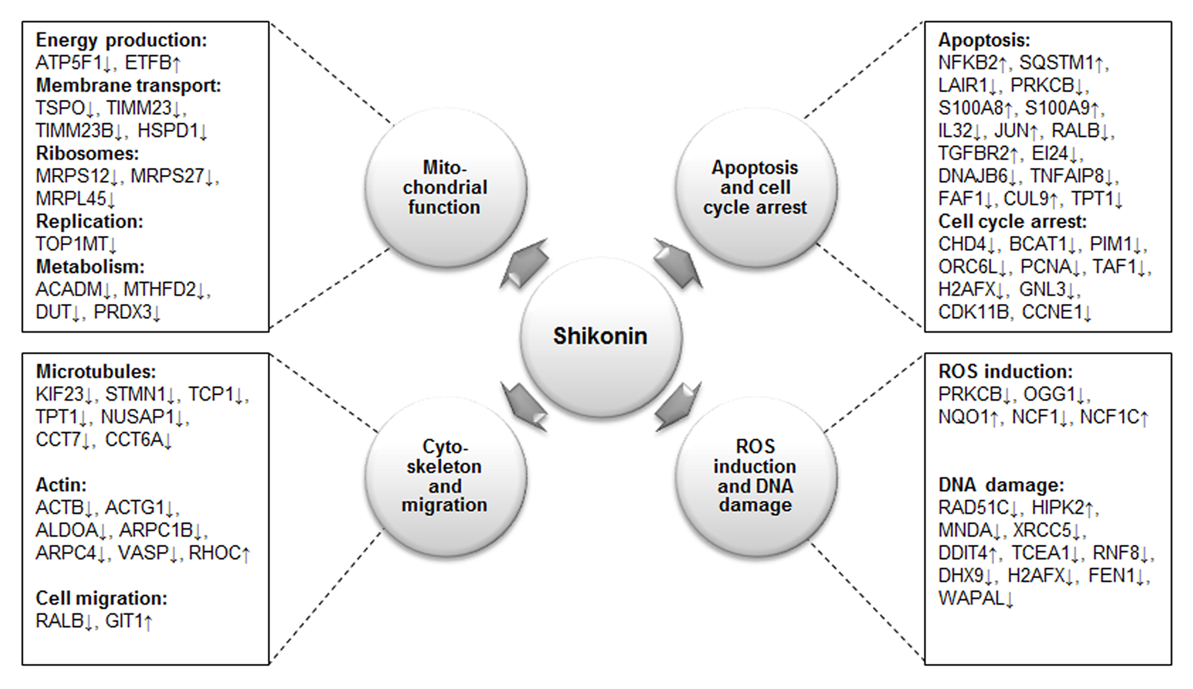


**Supplementary Figure S1: Summary of shikonin-affected mechanisms in U937 cells.** Examples of deregulated genes important for each mechanism are shown laterally in the boxes. Arrows pointing downwards (↓) or upwards (↑) indicate down- or up-regulation of gene expression, respectively.

Supplementary Video S1:

**Supplementary Video S1: Live imaging of the treatment of U2OS-GFP-αTubulin cells stably transfected with a GFP fusion construct of α-tubulin with 25 µM shikonin.** With increasing cellular concentrations of shikonin, the number of distinct tubulin filaments decreased and the tubulin staining became progressively diffuse.

Supplementary Video S1:

**Supplementary Video S2: Live imaging of the treatment of RPE-1-GFP-EB3 cells stably expressing GFP-EB3 with 25 µM shikonin.** Shikonin caused a slowdown and finally a complete disappearance of EB3 particles within 3 min of application, indicating disrupted microtubule formation.
